# Supplementary material for: Is There a Correlation between Multiparametric Assessment in Ultrasound and Intrinsic Subtype of Breast Cancer?
Source: J Clin Med. 2021 Nov 19;10(22):5394. doi: 10.3390/jcm10225394 (PMC8618837; doi:10.3390/jcm10225394)
Supplement: Supplementary file 1 [file jcm-10-05394-s001.zip › jcm-1436468-supplementary.pdf]

Supplementary Materials

Material S1. Methods

In our study four molecular biomarkers were used in patients with invasive breast carcinoma (IBC): estrogen receptors (ER), progesterone receptors (PR), HER2 and Ki-67.

Antibodies for ER , PR, Ki-67 (Daco Omnis company, according protocol Dako company, nuclear reaction) were used. For HER2 antibodies (Ventana company, staining on Benchmark machine, membrane reaction) were used. ER, PR were considered positive if tumors have 1% or more than nuclear-stained cells. HER2 staining was scored on a scale 0 to 3+according to the guidelines. The cut off for Ki-67 ≥14%was considered as high proliferation index (range from 0-to 100).

ER-is activated by the estrogen hormone, present in normal and IBC cells. Estrogen hormone stimulates the growth of normal breast epithelial cells and in IBC. ER are measured in BC tissue in our study using immunohistochemistry methods. It is performed on routinely prepared histological samples, primarily formalin-fixed paraffin-embedded (FFPE) tissue sections. Then slides are assessed microscopically to determine the intensity and proportions of positive cells. ER positive tumors are when ≥1%positive -staining tumor cells.

PR-is also routinely assessed similar to ER by immunohistochemistry methods, and is inactivated by progesterone hormone and stimulate the growth of tumor cells. PR positive tumors are positive, when ≥1% staining tumor cells is observed. The phenotype ER/PR positive is most frequent type of BC [1]

Expression of ER and PR determines the responsiveness of tumors to hormone therapy.

HER2 is a growth factor receptor, which is associated with cell proliferation, development and survival .The over-expression of HER2 status indicates a poorer prognosis. Today number of HER2-targeted therapies are available improve the overall survival. Is located on the surface at low levels in normal breast epithelium [2].

Table S1. Inferential results of univariate logistic regression explaining individual molecular cancer subtypes with US features.

	LA	LB HER2−	LB HER2+	HER2+	TNBC
Shape					
Regular	OR = 4.387 p = 0.2345 CI: [0.4057–96.5053]	NA *	NA *	OR = 2.8 p = 0.4125 CI: [0.1255–31.0646]	NA *
Irregular	Ref	Ref	Ref	Ref	Ref
Orientation					
Parallel	OR = 0.35 p = 0.0672 CI: [0.1123–1.0816]	OR = 3.155 p = 0.2836 CI: [0.5677–59.2076]	OR = 3.155 p = 0.2836 CI: [0.5677–59.2076]	OR = 2.917 p = 0.3186 CI: [0.5217–54.833]	OR = 0.573 p = 0.3909 CI: [0.1688–2.283]
Non-parallel	Ref	Ref	Ref	Ref	Ref
Margin					
Circumscribed	NA *	NA *	NA *	NA *	NA *
Indistinct	Ref	Ref	Ref	Ref	Ref
Angular/ spiculated	OR = 0.977 p = 0.9615 CI: [0.3806–2.6144]	OR = 1.466 p = 0.5404 CI: [0.4603–5.64]	OR = 1.193 p = 0.7807 CI: [0.3644–4.6599]	OR = 2.315 p = 0.301 CI: [0.557–15.791]	OR = 0.389 p = 0.088 CI: [0.1298–1.1689]
Micro/macrolobulated	OR = 1.000 p = 1 CI: [0.1785–4.7934]	NA *	OR = 1.643 p = 0.6079 CI: [0.1978–10.4917]	OR = 3.571 p = 0.2418 CI: [0.3746–34.5553]	OR = 0.679 p = 0.6686 CI: [0.0881–3.6026]
Echo pattern					
Complex/hypoechoic	Ref	Ref	Ref	Ref	Ref
Hyper/isoechoic	OR = 2.241 p = 0.2764 CI: [0.4992–10.0823]	OR = 0.696 p = 0.7431 CI: [0.036–4.312]	OR = 0.696 p = 0.7431 CI: [0.036–4.312]	OR = 1.905 p = 0.457 CI: [0.2613–9.2842]	NA *
Posterior features					
No Posterior Features	Ref	Ref	Ref	Ref	Ref
Enhancement	NA *	OR = 0.323 p = 0.3262 CI: [0.0159–2.3083]	OR = 2.091 p = 0.4099 CI: [0.3393–12.9903]	OR = 5.750 p = 0.0324 CI: [1.2257–32.8005]	OR = 4.800 p = 0.0967 CI: [0.8049–38.8888]
Shadowing	OR = 0.368 p = 0.0911 CI: [0.1108–1.1472	OR = 0.764 p = 0.7144 CI: [0.1687–3.2667]	OR = 3.407 p = 0.1006 CI: [0.8497–17.3023]	OR = 0.639 p = 0.6402 CI: [0.0786–4.1934]	OR = 2.857 p = 0.2373 CI: [0.5519–21.4375]
Combined Pattern	OR = 0.565 p = 0.2759 CI: [0.1994–1.5725]	OR = 1.014 p = 0.9832 CI: [0.284–3.8394]	OR = 0.697 p = 0.6748 CI: [0.1196–4.0566]	OR = 1.237 p = 0.7856 CI: [0.2747–6.5233]	OR = 3.429 p = 0.1415 CI: [0.7685–24.1953]
Calcifications					
Present	OR = 0.444 p = 0.0604 CI: [0.1866–1.0261]	OR = 2.457 p = 0.1178 CI: [0.8332–8.2791]	OR = 3.488 p = 0.0411 CI: [1.1305–13.1713]	OR = 3.143 p = 0.0632 CI: [1.0054–11.9458]	OR = 0.176 p = 0.0041 CI: [0.0469–0.5335]
Absent	Ref	Ref	Ref	Ref	Ref

Additional features					
Skin changes	OR = 0.422<br>$p = 0.1518$<br>CI: [0.1132–1.2689]	OR = 2.545<br>$p = 0.1083$<br>CI: [0.776–7.853]	OR = 2.545<br>$p = 0.1083$<br>CI: [0.776–7.853]	OR = 0.504<br>$p = 0.391$<br>CI: [0.0749–2.0193]	OR = 0.677<br>$p = 0.5681$<br>CI: [0.1461–2.3215]
Edema	OR = 1.422<br>$p = 0.4492$<br>CI: [0.583–3.6727]	OR = 0.63<br>$p = 0.3968$<br>CI: [0.2171–1.9027]	OR = 1.179<br>$p = 0.7766$<br>CI: [0.3944–4.0026]	OR = 0.557<br>$p = 0.2928$<br>CI: [0.1872–1.7084]	OR = 1.425<br>$p = 0.5343$<br>CI: [0.4893–4.7721]
Vascularity	OR = 0.255<br>$p = 0.0734$<br>CI: [0.0494–1.1078]	NA *	OR = 1.436<br>$p = 0.7431$<br>CI: [0.2319–27.8049]	OR = 1.329<br>$p = 0.7969$<br>CI: [0.2135–25.7841]	OR = 1.658<br>$p = 0.646$<br>CI: [0.2701–32.007]
Elastography					
Soft	Ref	Ref	Ref	Ref	Ref
Intermediate	OR = 0.522<br>$p = 0.3111$<br>CI: [0.1448–1.8523]	OR = 2.167<br>$p = 0.4994$<br>CI: [0.3077–43.6893]	OR = 2.690<br>$p = 0.3815$<br>CI: [0.4013–53.5133]	OR = 0.222<br>$p = 0.1237$<br>CI: [0.0265–1.5039]	OR = 2.400<br>$p = 0.3033$<br>CI: [0.5239–17.2288]
Hard	OR = 0.359<br>$p = 0.0977$<br>CI: [0.1043–1.2188]	OR = 3.405<br>$p = 0.2617$<br>CI: [0.5758–65.1953]	OR = 3.023<br>$p = 0.3126$<br>CI: [0.5047–58.1122]	OR = 0.960<br>$p = 0.956$<br>CI: [0.2459–4.7758]	OR = 0.913<br>$p = 0.9162$<br>CI: [0.1897–6.6557]

LA= luminal A; LB HER2- = luminal B without HER2 overexpression; LB HER2+ = luminal B with HER2 overexpression; HER2+ = human epidermal growth factor receptor 2 positive; TNBC = triple negative breast cancer. OR=Odds ratio, CI= Confidence interval, Ref=reference, NA*= not applicable

Supplementary References:

1. WHO Classification of tumours, 5th edition Breast tumours. Invasive breast carcinoma. *Gen. Overv. IARC Fr.* **2019**,*2*, 82–101.
2. O Nielsen, T.; Leung, S.C.Y.; Rimm, D.L.; Dodson, A.; Acs, B.; Badve, S.; Denkert, C.; Ellis, M.J.; Fineberg, S.; Flowers, M.; et al. Assessment of Ki67 in Breast Cancer: Updated Recommendations From the International Ki67 in Breast Cancer Working Group. *J. Natl. Cancer Inst.* **2021**, *113*, 808–819, doi:10.1093/jnci/djaa201.
